# Landscape of cardiorenal syndrome research: A bibliometric analysis

**DOI:** 10.1097/MD.0000000000040558

**Published:** 2024-11-29

**Authors:** Yan Sun, Bin Hua, Yan Bai, Wang Zheng, Lin Shen, Wangkang Niku, Yihong Wei, Fan Zhang, Bing Deng

**Affiliations:** a Department of Cardiology, Longhua Hospital Shanghai University of Traditional Chinese Medicine, Shanghai, China; b Department of Nephrology A, Longhua Hospital Shanghai University of Traditional Chinese Medicine, Shanghai, China.

**Keywords:** bibliometric analysis, cardiorenal syndrome, cardiovascular disease, citation, VOSviewer

## Abstract

To comprehensively review cardiorenal syndrome (CRS)-related research, this study analyzes its whole landscape and future trends from a bibliometric perspective. Using “cardiorenal syndrome” as the key word, a representative dataset of CRS was constructed in the Web of Science Core Collection (Science Citation Index Expanded) database up to November 2023. VOSviewer (version 1.6.18) and R-Studio (version 1.4) were used to visualize CRS-related author and national collaboration networks, hotspot distribution clustering and historical citation networks. The annual number of publications shows an upward trend, especially since 2009. The United States is the most active country and closely cooperates with European countries. *Cardiorenal Medicine* is the journal that publishes the most CRS-related research. Urology & Nephrology and Cardiovascular System & Cardiology are the most prominent areas of CRS literature output. The leaders in CRS-related research are Ronco C from Italy and Mccullough PA, from the United States. Combined with keyword co-occurrence, theme evolution, and strategic distribution analysis, it was found that centering on the mechanism of CRS (cardiovascular lesions, venous congestion, and fibrosis), prognosis (transplantation, dialysis, hospitalization, mortality) and cardiac function management (b-type natriuretic peptide, diastolic dysfunction, left ventricular hypertrophy) is a possible research direction for a long time to come. Although CRS has a relatively short history, it has received a lot of attention and is currently enjoying a high level of overall acceptance. In conclusion, this study explores the major research areas, frontiers and trends in CRS, which are important for future research.

## 1. Introduction

It is well known that cardiovascular disease and chronic kidney disease share mechanisms that influence each other’s deterioration.^[1,2]^ Patients with cardio-renal co-morbidities are not only at significantly higher risk of cardiovascular events but also have an equally significant increased risk of cardiovascular and all-cause mortality.^[3–5]^ The Atherosclerosis Risk in Communities study found that compared to controls with an estimated glomerular filtration rate ≥ 90 mL/min/1.73 minutes^2^, the incidence of heart failure in those with an estimated glomerular filtration rate < 60 mL/minutes/1.73 m^2^ had a 3-fold higher incidence of heart failure (18 vs 6/1000 person-years).^[6]^ Among them, a significantly greater decline in renal function was observed in heart failure hospitalized/death compared with those who did not develop heart failure.^[6]^

Cardiac insufficiency and renal failure are usually causally related to apoplexy. In this context, cardiorenal syndrome (CRS) emerged, a clinical syndrome in which acute or chronic dysfunction of 1 organ triggered by cardiac or renal disease induces acute or chronic dysfunction of the other organ.^[7]^ In recent years, along with the dramatic increase in the number of patients with cardiovascular disease and chronic kidney disease, it has received extensive attention, both in terms of CRS staging, prognosis, pathophysiology, clinical evaluation, and diagnosis and treatment.^[8–10]^

Bibliometrics analysis is the qualitative and quantitative evaluation of specific research areas using mathematical and statistical methods to understand the knowledge structure and explore development trends.^[11]^ The process allows the comparison of contributions and collaborations across authors, countries, and journals. Further, bibliometric analysis can predict the hotspots and trends within a specific research area through information visualization.^[12]^

Based on literature in the Web of Science Core Collection database, this study aims, through bibliometric analysis, to reveal global trends in CRS-related research; explore core journals, authoritative authors, and research topics in CRS; provide valuable insights into CRS-related research.

## 2. Methods

### 2.1. Search strategy

The Web of Science Core Collection database (Science Citation Index Expanded [SCI-Expanded]) was selected as a data source, corresponding to the following search strategy: TS= (“Cardio Renal Syndrome” OR “Cardio-Renal Syndromes” OR “Reno-Cardiac Syndrome” OR “Reno Cardiac Syndrome” OR “Reno-Cardiac Syndromes” OR “Renocardiac Syndrome” OR “Renocardiac Syndromes” OR “Cardiorenal Syndrome” OR “Cardiorenal Syndromes”). All records were exported as “plain text file” or “Tab delimited file” with “Full Record and Cited References.” Ethical approval was not required as the study was a secondary study.

### 2.2. Bibliometric analysis

**Figure 1.**
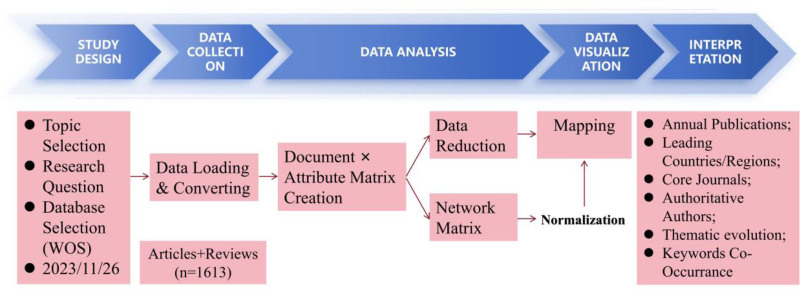
Workflow for science mapping.

This study follows the 5-step bibliometric approach proposed by Aria and Cuccurullo.^[13]^: study design, data collection, data analysis, data visualization, and interpretation. Figure 1 is a schematic representation of the entire methodology. First, we selected “cardiorenal syndrome” as the research topic in the study design phase and identified 3 research questions. Second, we searched the Web of Science Core Collection (SCI-Expanded) database and obtained 2102 publications (26 November, 2023). We restricted documents to articles and reviews because the peer review process facilitates reliable scientific communication, stimulates meaningful research questions, and ensures the accuracy of conclusions.^[14]^ The final sample consisted of 1613 documents published between 1977 and 2023. All records were imported into the biblioshiny web program^[13]^ and VOSviewer software^[15]^ to draw concept maps, co-citation network diagrams, and other graphs. The overall overview of this bibliometrics is shown in Figure S1, Supplemental Digital Content, http://links.lww.com/MD/O62.

### 2.3. Ethics statement

No ethical approval is required because this study is a bibliometric analysis.

## 3. Results

### 3.1. Annual publications

**Figure 2.**
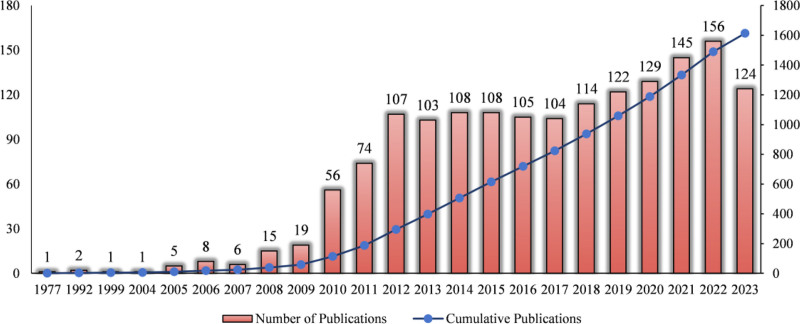
Annual publications of CRS-related literature from 1977 to 2023. CRS = cardiorenal syndrome.

Figure 2 shows the scientific results from 1977 to 2023. The first paper in the Web of Science Core Collection (SCI-Expanded) database on CRS was published in 1977 as a case report entitled “Facio-cardio-renal syndrome: a newly delineated recessive disorder.” Starting with only 1 paper published that year, annual publications grew slowly. After 2009, the number of CRS-related publications increased rapidly, reaching 156 by 2022, with a yearly growth rate of 11.27%. On average, 70 CRS-related research papers are published annually, with an average of 23.44 citations per paper.

### 3.2. Leading countries/regions

**Figure 3.**
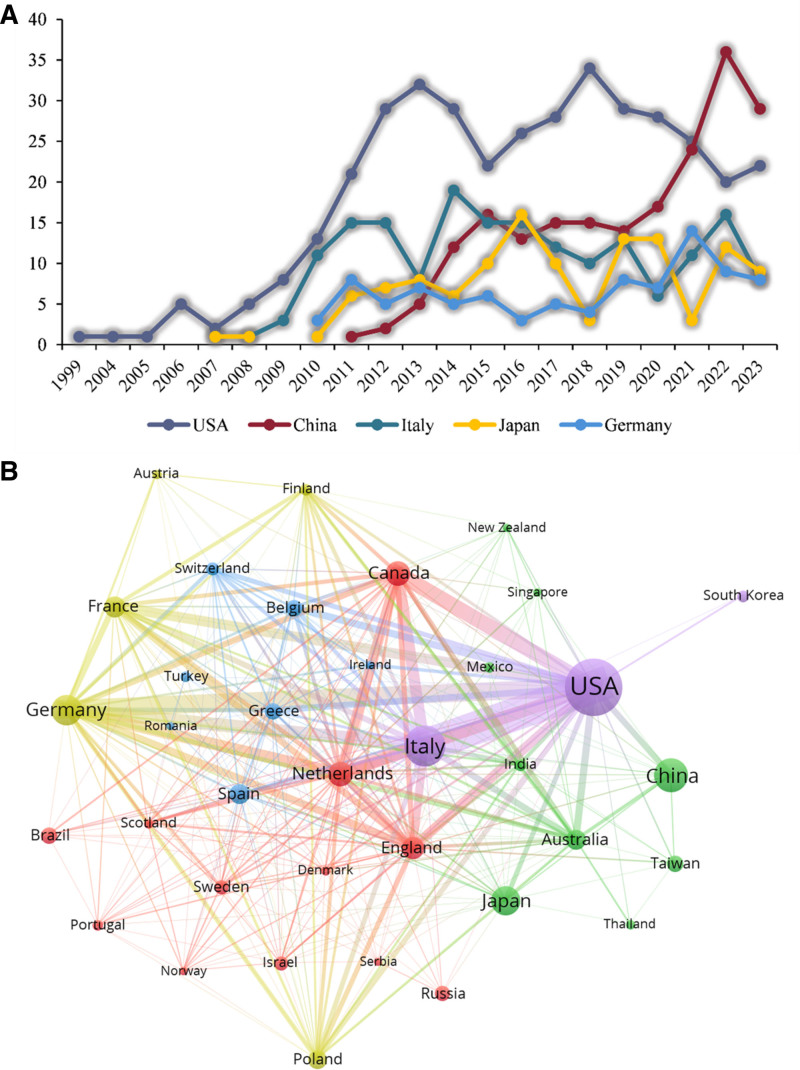
(A) Top 5 countries according to annual production. (B) Country cooperation network mapping.

The results show that 69 countries participated in the CRS study. The top 5 countries with the highest research outputs were accessed through Bibliometrix. The United States takes the lead, with China proliferating to surpass the United States in the number of papers published since 2021 (Fig. 3A). China issued its first CRS research results in 2011, which has increased yearly, reaching 38.7% by 2022 (Fig. 3A).

Figure 3B shows the current collaboration status between the thirty most productive countries/regions globally. The larger the country node, the more relevant research is conducted, and the links between countries represent partnerships. Overall, there are also more collaborations between countries with many publications, with the United States being the most prominent, working mainly with Canada, France, Germany, Italy, the Netherlands, and the United Kingdom (Fig. S2, Supplemental Digital Content, http://links.lww.com/MD/O62). The United States also leads in citations, but the country with the highest average article citations is the Netherlands (Fig. S3, Supplemental Digital Content, http://links.lww.com/MD/O62).

### 3.3. Core journals

**Figure 4.**
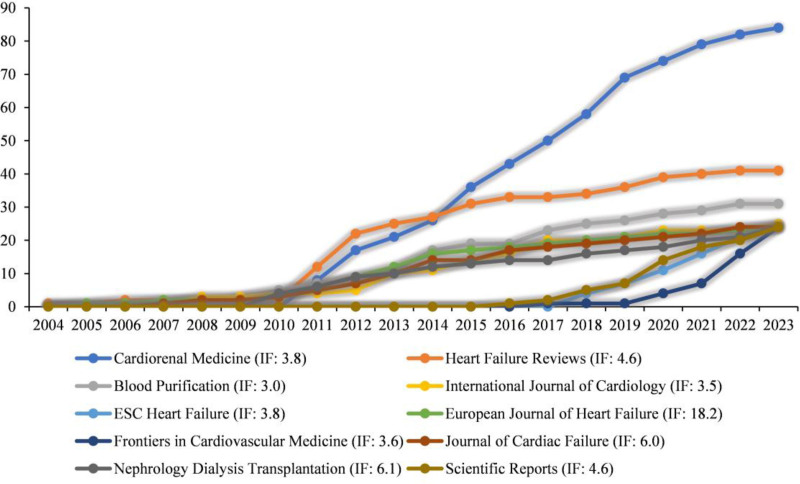
Temporal analysis of publication journals of CRS-related research. CRS = cardiorenal syndrome.

CRS studies were published in a total of 457 journals. As can be seen in Figure 4, the top 3 journals in terms of publications were *Cardiorenal Medicine* (n = 84), *Heart Failure Reviews* (n = 41), and *Blood Purification* (n = 31). The journal with the highest annual growth rate in publications was *Cardiorenal Medicine*, with 3 journals having an impact factor of more than 5.

The 10 most influential journals were selected based on the total citations, as shown in Table 1. The journals marked with an asterisk are the core journals in the CRS research area under Bradford’s Law, including *Journal of the American College of Cardiology*, *European Journal of Heart Failure*, *Nephrology Dialysis Transplantation*, and *Journal of Cardiac Failure*. Thus, these journals played a crucial role in CRS research.

**Table 1:** Top 10 journals ranked by total citations in CRS research.

Journals	Total citation	2023 IF
*Journal of the American College of Cardiology* *	5359	24
*Circulation*	4215	37.8
*New England Journal of Medicine*	3320	158.5
*Kidney International*	3280	19.6
*Journal of the American Society of Nephrology*	2733	13.6
*European Heart Journal*	2498	39.3
*European Journal of Heart Failure* *	2259	18.2
*Nephrology Dialysis Transplantation* *	1885	6.1
*Journal of Cardiac Failure* *	1774	6
*American Journal of Kidney Diseases*	1595	13.2

* Bradford’s Law reveals journal distribution to identify influential sources.

CRS = cardiorenal syndrome.

### 3.4. Category analysis

**Figure 5.**
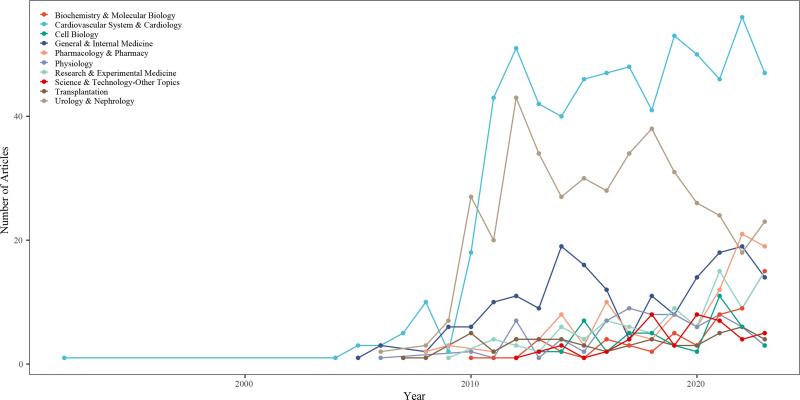
Temporal evolution of top 10 most productive Web of Science research areas in CRS-related literature. CRS = cardiorenal syndrome.

Web of Science research areas assigned by Clarivate Analytics were used to categorize the included literature, with each paper falling into at least 1 research area. In this study, the number of research areas covered by CRS literature increased from 1 in 1977 to 33 in 2023 (Fig. S4, Supplemental Digital Content, http://links.lww.com/MD/O62). The top ten most productive research areas are Biochemistry & Molecular Biology, Cardiovascular System & Cardiology, Cell Biology, General & Internal Medicine, Pharmacology & Pharmacy, Physiology, Research & Experimental Medicine, Science & Technology-Other Topics, Transplantation, Urology & Nephrology, accounting for 1479 of the 1613 papers, or about 91.69% of the total. Figure 5 shows the yearly evolution of the top 10 most productive areas of CRS research, illustrating the trajectory of CRS research focus areas. Before 2008, the most dominant study area was Cardiovascular System & Cardiology, with Urology & Nephrology, which rose rapidly in 2009 to 2010 and then became the most dominant CRS literature output area with Cardiovascular System & Cardiology.

### 3.5. Authoritative authors

The h-index is calculated based on the number of citations a scientist receives and is a widely accepted measure of scientific performance. The top ten authors with the highest h-index are Ronco C (37), Mccullough PA (20), Tang WHW (19), Cruz DN (18), and Vescovo G (18; Table 2). Ronco C is the most cited researcher in the Web of Science database. Ronco C and Mccullough PA are leaders in CRS-related research. Half of the top 10 authors is from Italy, 3 are from Germany, and the other 2 are from Chile and Australia, respectively.

**Table 2:** Top ten most influential authors ranked by the h-index.

Element	h-index	Number of publications	Country
Ronco C	37	120	Italy
Mccullough PA	20	37	USA
Tang WHW	19	29	USA
Cruz DN	18	25	USA
Vescovo G	18	27	Italy
Anker SD	17	20	Germany
Virzì GM	16	28	Italy
Braam B	15	21	Canada
Palazzuoli A	15	22	Italy
Testani JM	15	26	USA

**Figure 6.**
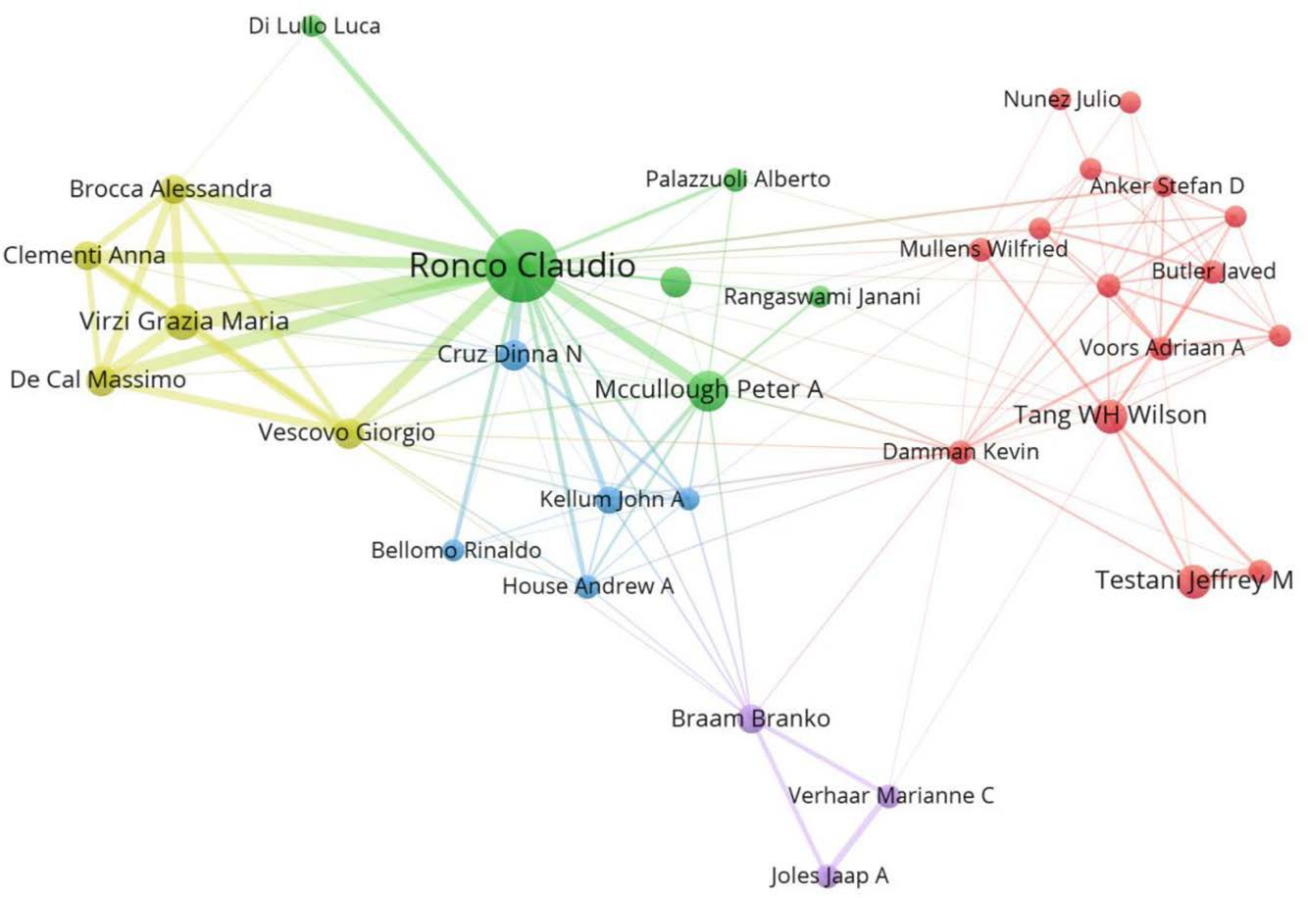
Author collaborative network mapping.

The average number of coauthors per paper was 7.23, and the percentage of international co-authorship reached 26.04%. This result suggests that CRS is a typical field of multi-authored collaborations. VOSviewer identifies 42 authors who have collaborated at least ten times, and the collaboration network mapping shows 5 representative collaborative teams centered on Ronco C, which bridges the entire author collaboration network (Fig. 6).

### 3.6. CRS highly cited literature

Table 3 lists the top 10 most cited literature in the field of CRS. For citation analysis, we extracted the average annual citations for the article. This value can be used as a proxy for the total citations to assess the current impact of a report. Papers with high total citations but low annual citations are more likely to be historically essential and may not reflect present influence. The average of these classic citations is 580.5 ± 325.2, the average per year is 67.4 ± 38.4, and they are an indispensable foundation and groundwork for CRS-related studies. Breaking down CRS-related topical studies by year and linking to their internal citation networks demonstrates the development of CRS (Fig. S5, Supplemental Digital Content, http://links.lww.com/MD/O62).

**Table 3:** Top 10 cited CRS-related studies.

Rank	Title	First author	Year	Journal	Total citations	Annual citations
1	Cardiorenal syndrome	Ronco Claudio	2008	*Journal of the American College of Cardiology*	1408	88
2	Acute kidney injury	Ronco Claudio	2019	*Lancet*	763	152.6
3	Cardio-renal syndromes: Report from the consensus conference of the Acute Dialysis Quality Initiative	Ronco Claudio	2010	*European Heart Journal*	641	45.79
4	Ultrafiltration in decompensated heart failure with cardiorenal syndrome	Bart Bradley A	2012	*New England Journal of Medicine*	594	49.5
5	Cardiorenal Syndrome: classification, pathophysiology, diagnosis, and treatment strategies: A scientific statement from the American Heart Association	Rangaswami, Janani	2019	*Circulation*	503	100.6
6	The outcome of neutrophil gelatinase-associated lipocalin-positive subclinical acute kidney injury: A multicenter pooled analysis of prospective studies	Haase Michael	2011	*Journal of the American College of Cardiology*	496	38.15
7	2017 Comprehensive update of the Canadian Cardiovascular Society Guidelines for the management of heart failure	Ezekowitz Justin A	2017	*Canadian Journal of Cardiology*	405	57.86
8	Cardiorenal Syndrome new perspectives	Bock Jeremy S	2010	*Circulation*	343	24.5
9	Slow recovery from critical Coronavirus disease 2019 pneumonia in an immunosuppressed renal transplant recipient with early acute cardiorenal syndrome	Zhu Lan	2020	*Cardiorenal Medicine*	327	81.75
10	Heart failure and kidney dysfunction: epidemiology, mechanisms and management	Schefold Joerg C	2016	*Nature Reviews Nephrology*	325	40.63

CRS = cardiorenal syndrome.

### 3.7. Trends in CRS-related research and themes distribution

**Figure 7.**
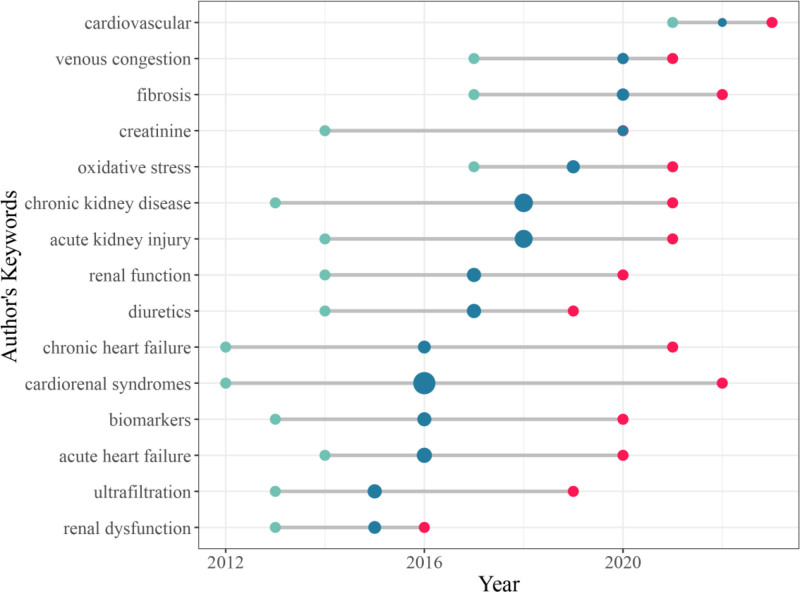
Temporal trends in author keywords. Note: The X-axis represents the year, and the Y-axis represents the keywords. The position of the green dot is the first quartile of the publication year corresponding to the keyword, the position of the red dot is the third quartile of the publication year, and the position of the blue dot is the median of the publication year, and the size of the bubble reflects the number of papers.

Figure 7 shows the trend of CRS-related research hotspots over time, which can reflect the thematic evolution of CRS research. The span of the horizontal line indicates the period of word outbreak, and the size of the shadow ball means the word frequency. Undoubtedly, the words with the most prolonged sustained attention are cardiorenal syndromes, chronic heart failure/kidney disease, and acute heart failure/ kidney disease. Among these keywords, cardiovascular, venous congestion, and fibrosis have become the last 2 years’ essential research directions.

**Figure 8.**
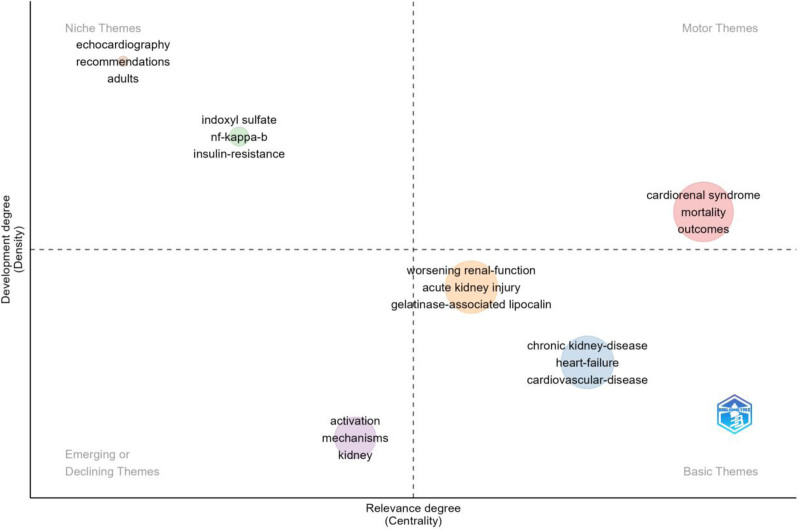
Strategic distribution of CRS-related research concerns. CRS = cardiorenal syndrome.

Figure 8 shows the distribution and future trends of CRS-related studies in the form of strategic coordinates. Such as the mortality risk, prognosis, deterioration of renal function, acute kidney injury, and gelatinase-associated lipocalin in CRS have developed significantly, and they need to be further explored while promoting the development of CRS-related research.

### 3.8. Research hotspots and keyword co-occurrence

**Figure 9.**
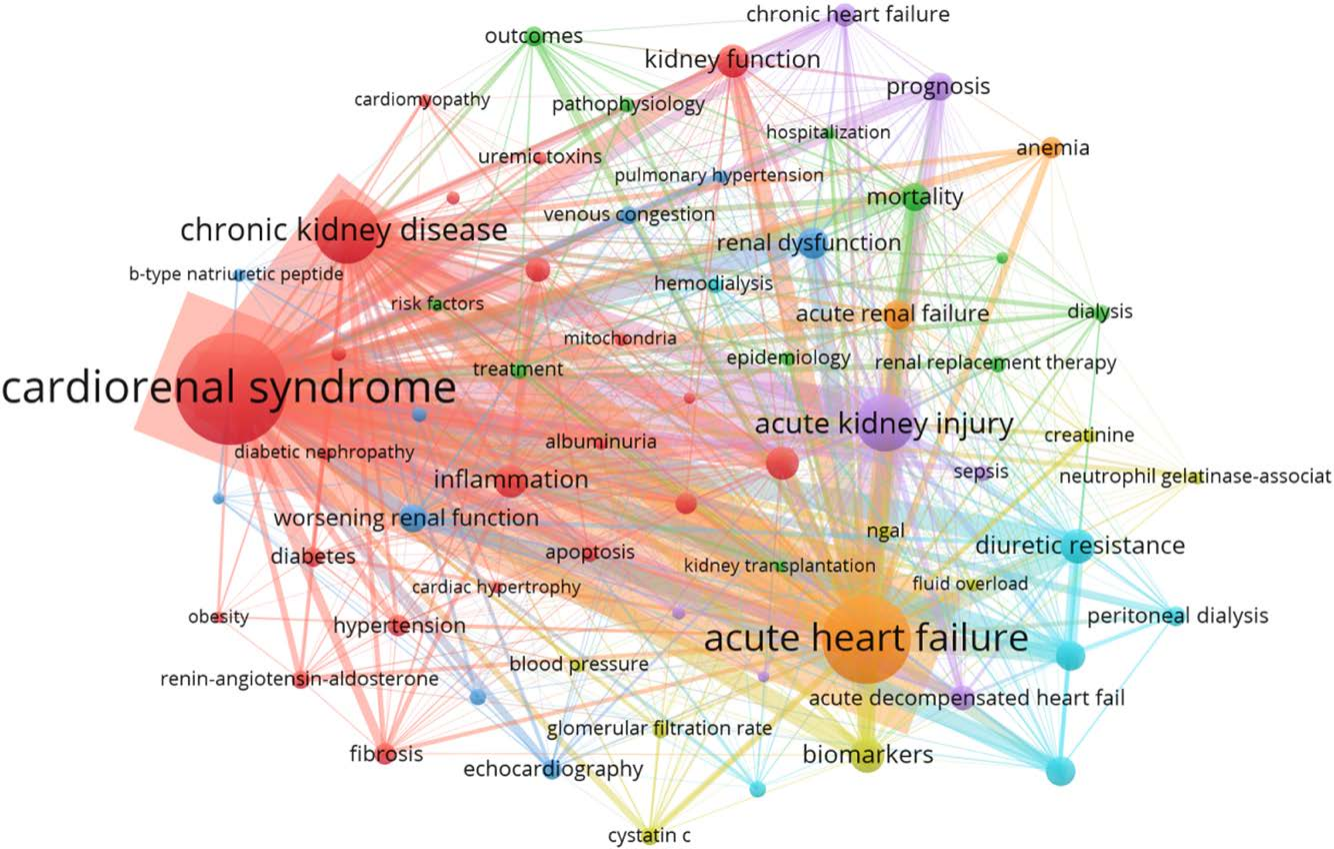
Keyword co-occurrence network mapping.

The keyword co-occurrence network reveals the hottest content in the field of CRS. Of the 2340 author keywords, 71 had a frequency of occurrence of 10 or more. VOSviewer categorized them into 6 clusters and formed network connections, revealing the main areas and directions of development (Fig. 9). Different colors distinguish different clusters, each representing a category of research topics. Cluster 1 is the largest (in red) and includes 22 keywords mainly related to cardiac hypertrophy, cardiomyopathy, fibrosis, inflammation, and oxidative stress, centering on the mechanism of CRS. Cluster 2 contains 11 keywords (in green), mainly on heart transplantation, kidney transplantation, dialysis, hospitalization, mortality, and risk factors, focusing on the treatment and prognosis of CRS. Group 3 focuses on individual cardiac function, including b-type natriuretic peptide, diastolic dysfunction, and left ventricular hypertrophy (dark blue).

## 4. Discussion

### 4.1. General information

This study reviewed 1613 CRS-related literature and found that since 2009, there have been increasing studies on CRS, and CRS has gradually attracted more and more researchers’ attention. The annual publications and trends of a research topic can reflect the awareness and overall development of the field.^[16]^ With the 2008 expert consensus of the Acute Dialysis Quality Initiative^[7]^ clarifying the definition of CRS, researchers have paid more and more attention to the clinical problems of CRS, which is also the reason for the rapid growth of the number of literatures in this field.

As early as 1952, some scholars proposed the “prototype” of CRS on the phenomenon of kidney injury caused by heart failure; until 2005, Bongartz et al^[17]^ first proposed the concept of CRS, and then Ronco et al^[7]^ improved the idea at the 2008 Acute Dialysis Treatment Initiative conference and divided it into 5 subtypes according to its pathophysiological characteristics. Thus, a “cycle drama” about the heart and kidney has begun, and the clinical evidence around CRS has been enriched, promoting the construction of related guidelines.

The level of development of CRS in different countries varies widely, with Europe, North America, China, and Japan leading the way. The United States has published the most and the earliest papers in the field of CRS, followed by China and Italy, and although China first published CRS-related studies later, it surpassed the United States in terms of annual citations in 2022. Nonetheless, China’s corresponding total citations only ranked fourth, with average article citations of only 12.00, far behind Europe and the United States, reflecting the quality of research in Europe and the United States, which also drove the CRS research boom. Ronco C from the University of Padua, Italy, is one of the primary authors of CRS, who has an excellent co-presentation in the field of renal replacement therapy, covering renal pathophysiology, renal replacement therapy techniques, and management of renal failure, and whose research results are of great significance in advancing the development of nephrology and improving clinical practice. Another cardiologist from the University of Texas School of Medicine, Mccullough PA, whose research work in heart and kidney is outstanding, focuses on the interrelationship between cardiovascular disease and renal function, and especially on the cross-impact between heart failure and renal disease, has made significant contributions.

Regarding sources, *Cardiorenal Medicine* is the journal that publishes the most CRS-related research (40.1%), founded in 2011 by Sowers JR. *Cardiorenal Medicine* explores how obesity and other metabolic abnormalities promote the pathogenesis and progression of heart and kidney disease (i.e., cardiorenal metabolic syndrome). It provides an interdisciplinary platform for advancing research and clinical practice, focusing on translational issues.^[18]^ Regarding citations, studies published in the *Journal of the American College of Cardiology* are the journals researchers focus on and cite. There is no doubt that the *Journal of the American College of Cardiology* is one of the most influential journals in the field of cardiovascular medicine worldwide. Indeed, the CRS-related studies published in the *Journal of the American College of Cardiology* have contributed to the development and progress of the field of CRS.

The yearly evolution of the ten most productive categories illustrates the changing areas of research focus in CRS. Before 2009, the predominant research area in CRS was the cardiovascular system; Urology & Nephrology rose rapidly in later years with the publication of the CRS staging guidelines in 2008, but research momentum has waned in the last 5 years. Meanwhile, with the publication of several large clinical trials on sodium-dependent glucose transporters 2 inhibitors and the novel nonsteroidal saline corticosteroid receptor antagonist finerenone, Pharmacology & Pharmacy has emerged as a research hotspot. The 2023 European Society of Cardiology Annual Meeting published the “2023 ESC Guidelines for the Management of Cardiovascular Disease in Patients with Diabetes”^[19]^ and the “2023 Focused Update of the 2021 ESC Guidelines for the Diagnosis and Treatment of Acute and Chronic Heart Failure,”^[20]^ for the first time, finerenone was included in the cardiovascular guidelines. Both were given the highest level of recommendation, further enhancing the concept and importance of cardiorenal co-treatment. Undoubtedly, these 2 novel classes of pharmacologic agents will continue to be research hotspots in CRS. In recent years, high-quality studies with chronic kidney disease have begun exploring novel drugs’ effects on cardiovascular endpoint events.^[21–23]^ Conversely, there have also been observations of the ameliorative effects of drugs on the kidneys and the elucidation of their protective effects on heart failure in large-scale randomized controlled studies with chronic heart failure as the study population.^[24]^

Highly cited literature, or citation classics, is usually the focus of researchers’ attention.^[25]^ The most frequent citations were received by Ronco et al^[7]^ in 2008, published in the *Journal of the American College of Cardiology*, “Cardiorenal syndrome,” which proposed a new CRS classification that reflects the pathophysiology, time frame, and nature of concurrent cardiac and renal dysfunction. Our breakdown of CRS-related studies by year and their internal citation weblinks illustrate the progression (Fig. S5, Supplemental Digital Content, http://links.lww.com/MD/O62). In 2004, Heywood^[26]^ reported on the causes of impaired renal function and therapeutic strategies in patients with heart failure; Bongartz et al^[17]^ in 2005 proposed that severe CRS is a pathophysiologic state in which a combined decline in the function of the heart and kidneys leads to an individual’s organ failure, which in turn increases cardiovascular morbidity and mortality; as well as the 2 articles published by Ronco et al^[7,27]^ in 2008, these 4 reports laid a solid foundation for the subsequent clinical practice and scientific research in CRS.

Since the concept of CRS was proposed in 2005, and a specific staging elaborated in 2008, only more than 15 years ago, there are relatively few existing studies on CRS, and the research hotspots are relatively concentrated. The focus is pathophysiologic and mechanistic studies, early diagnostic and predictive markers, molecular mechanisms of cardiorenal interactions, and clinical prognosis and risk assessment. In the short period after the concept of CRS was introduced, scholars have been exploring the pathophysiologic mechanisms of the interactions between the heart and kidney, including inflammation, metabolic disorders, and neuroendocrine system disorders. In recent years, with the maturation of genomics, transcriptomics, and proteomics, researchers have continued to delve deeper into the molecular mechanisms of heart-kidney interactions to provide more precise treatment strategies for patients. Therefore, the pathophysiological study of CRS will continue to receive more and more attention and will elaborate its pathogenesis with more specified mechanisms in the future. On the other hand, the results of several cohort studies have clarified the need for prognostic models and assessment tools for predicting patients with CRS, which may be based on various parameters such as clinical features, biochemical indices, and imaging tests.

Cardiovascular-related studies occupy the first place in CRS-related literature regarding the evolution of research themes. Circulation recently published “A Synopsis of the Evidence for the Scientific and Clinical Management of Cardiovascular-Kidney-Metabolic (CKM) Syndrome: A Scientific Statement From the American Heart Association”^[28]^ and “Cardiovascular-Kidney-Metabolic Health: A Presidential Advisory From the American Heart Association,”^[29]^ which introduced the new concept of cardio-renal metabolic syndrome, which focuses on more than just cardiovascular or chronic kidney disease, but moves the gateway forward, with cardio-renal metabolic syndrome focusing more on metabolic abnormalities on the heart/kidney. However, evidence-based medical evidence is available for glucagon-like peptide-1 receptor agonists and sodium-dependent glucose transporters 2 inhibitors that can safely control weight and glucose metabolism and improve cardiorenal regression outcomes. However, much remains unknown, so further in-depth research may lead to more refined strategies for preventing, treating, and managing cardiorenal disease.

The second-ranked keyword is venous congestion. It has been shown that venous congestion is a significant cause of renal insufficiency in patients with cardiorenal syndrome. Husain-Syed et al^[30]^ suggested that this renal disease phenotype should be named congestive nephropathy and proposed diagnostic criteria. Meanwhile, the team pointed out that the next step should be to elucidate the effects of prolonged venous congestion on renal pathology and function based on animal models, including immunohistological and ultrastructural studies. In clinical practice, despite the congested state of the kidneys, in patients with CRS, Husain-Syed et al^[30]^ suggested that renal biopsy should be considered to clarify the likelihood of recovery of renal function after decongestion. Studies are also needed to validate the role of renal Doppler ultrasonography in managing heart failure and pulmonary hypertension and assessing blood volume status.

### 4.2. Strength and limitations

Through bibliometric analysis, this study depicts the research dynamics (annual publications) of CRS from a macro perspective, reports the leading countries, authoritative authors, and core journals, as well as the thematic evolution of CRS-related research from a meso perspective, as well as investigates the research hotspots and future directions of CRS from a micro perspective. However, our study still has some potential limitations. First, this study only searched the literature in the Web of Science Core Collection database, and although it is one of the most authoritative scientific and technical literature search tools, it does not cover all the research on CRS. Second, bibliometric analysis is only an auxiliary tool, and the results may be biased from the real world; to minimize the discrepancy, we analyzed from different perspectives. Therefore, the results of this study are relevant. Third, to better visualize the keywords, we chose custom thresholds to limit their co-occurrence, possibly ignoring some recently emerged keywords.

## 5. Conclusion

CRS has significant research value and promise in both cardiovascular and renal diseases. Econometric analysis shows that CRS-related literature is growing gradually, and high-impact journals focus on this disease, indicating the importance of international scholars. The expanding research areas of CRS illustrate the complexity, and international cooperation tends to be in Europe and the United States. In the future, the mechanism of development and prevention around CRS will be the key direction.

## Author contributions

**Conceptualization:** Yan Sun, Bin Hua, Yan Bai, Wang Zheng, Lin Shen, Yihong Wei, Fan Zhang.

**Data curation:** Yan Sun, Bin Hua, Yan Bai, Wang Zheng, Lin Shen, Wangkang Niku, Yihong Wei, Fan Zhang.

**Formal analysis:** Yan Bai, Fan Zhang.

**Funding acquisition:** Bing Deng.

**Software:** Lin Shen.

**Writing – original draft:** Yan Sun, Bin Hua, Yan Bai, Wangkang Niku, Fan Zhang.

**Writing – review & editing:** Fan Zhang, Bing Deng.

## Supplementary Material


