# Supplementary material for: Landscape of cardiorenal syndrome research: A bibliometric analysis
Source: Medicine (Baltimore). 2024 Nov 29;103(48):e40558. doi: 10.1097/MD.0000000000040558 (PMC11608689; doi:10.1097/MD.0000000000040558)

Figure S1 Overview of included literature.


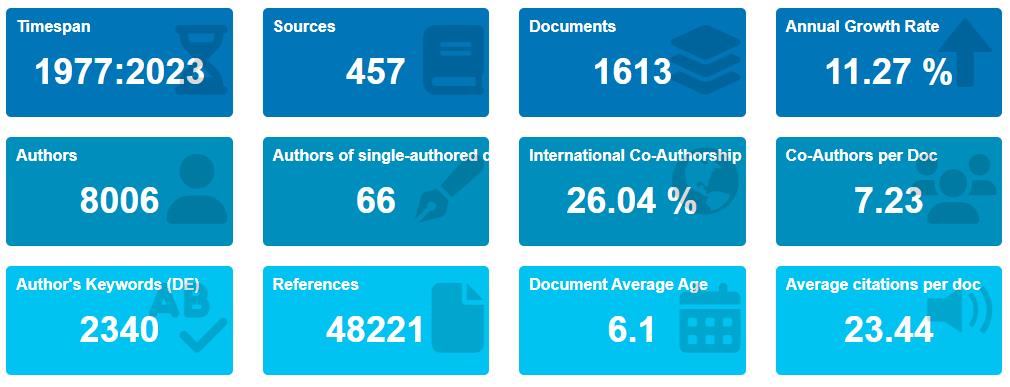


Figure S2 A mapping of the United States cooperative network


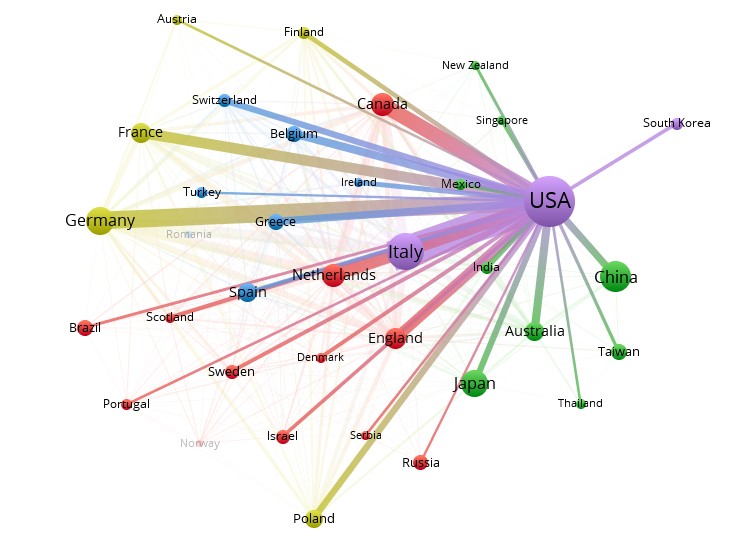


Figure S3 Top 10 country citations and average article citations


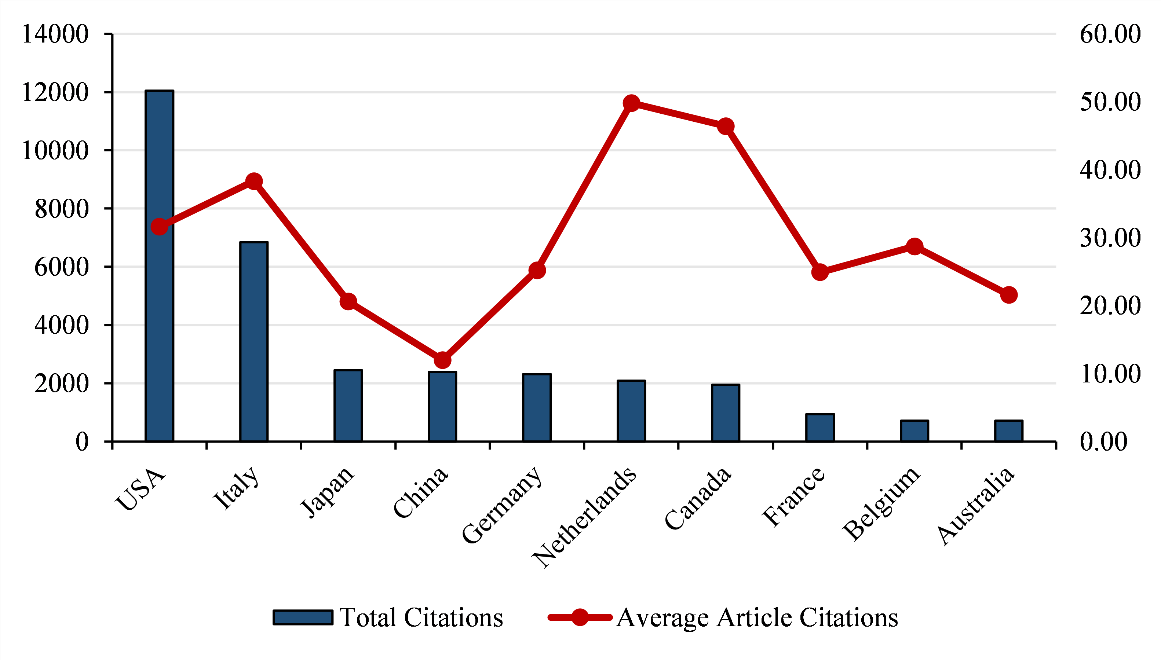


Figure S4 Number of Web of Science research areas covered in CRS-related literature


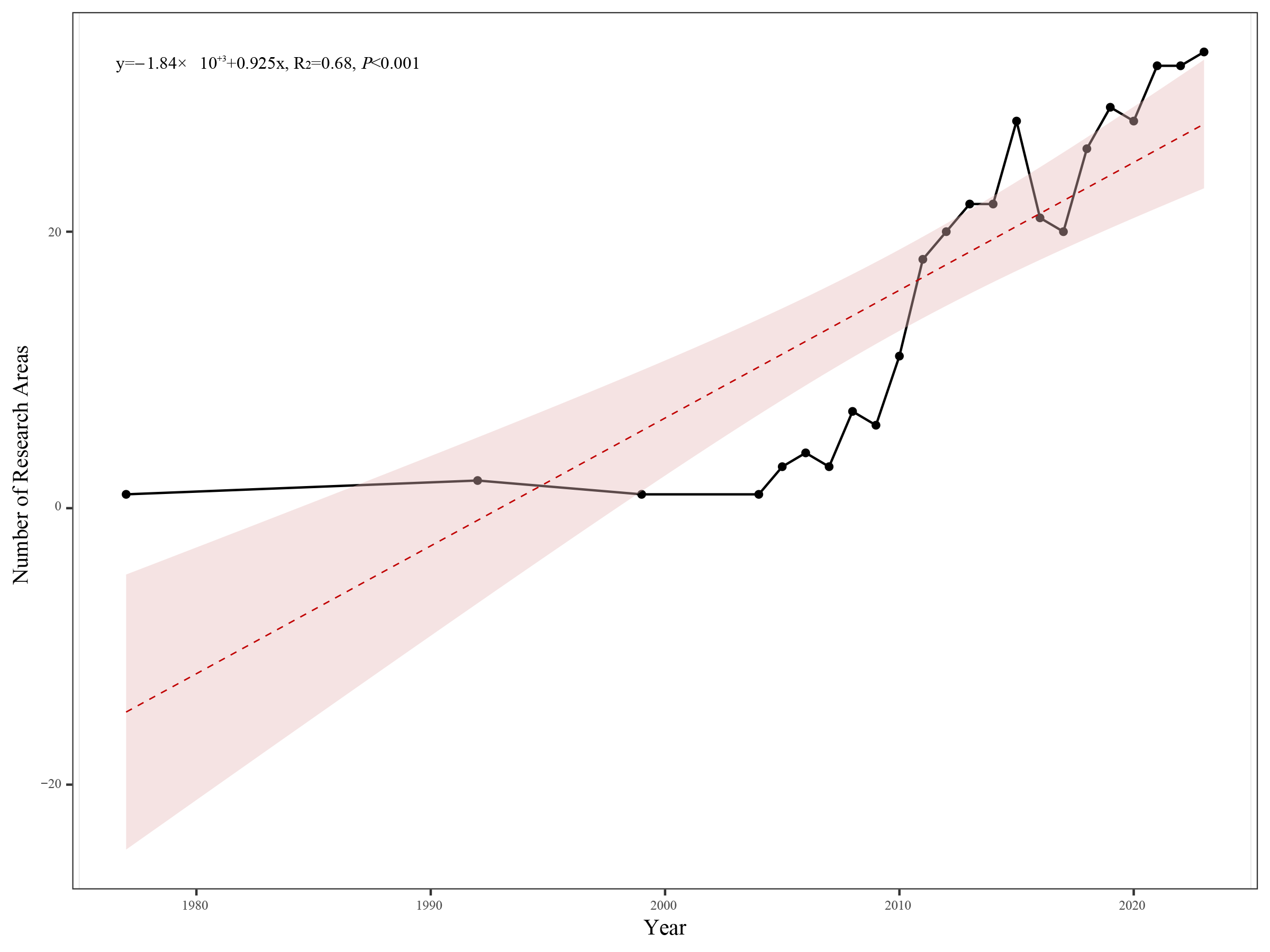


Figure S5 CRS-related research historical direct citation network.


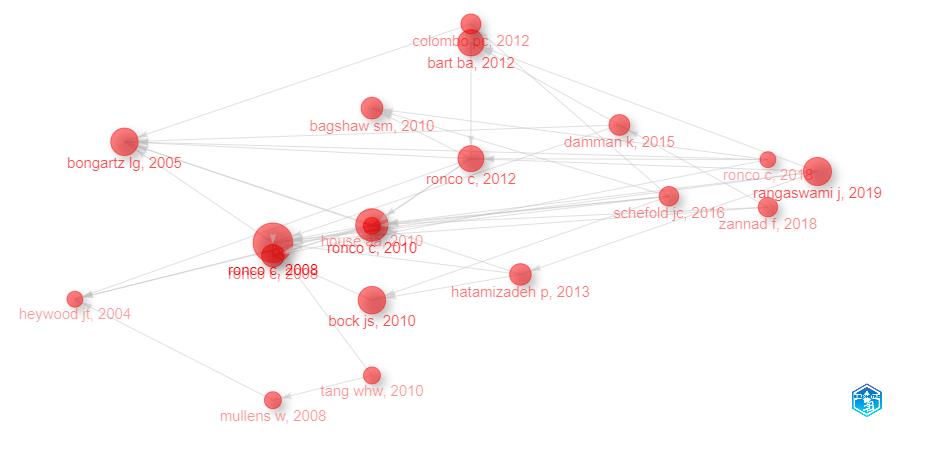

Supplement: Supplementary file 1 [file medi-103-e40558-s001.docx]
